# Functional and Radiological Outcomes of Distal Femur Fractures Fixed With Bicolumnar Plating: A Retrospective Study

**DOI:** 10.7759/cureus.95237

**Published:** 2025-10-23

**Authors:** Ashwin Kumar, Sagar Venkataraman, Nagakumar J S, Anil K Sakalecha, Gowtham Gandhi

**Affiliations:** 1 Orthopaedics, Sri Devaraj Urs Medical College, Sri Devaraj Urs Academy of Higher Education and Research, Kolar, IND; 2 Radio-Diagnosis, Sri Devaraj Urs Academy of Higher Education and Research, Kolar, IND

**Keywords:** bicolumnar plating, distal femur fracture, dual plating, knee society score, modified rust score

## Abstract

Background: Distal femur fractures are challenging orthopedic injuries, especially when involving the intra-articular extension or occurring in osteoporotic bone. With the increased use of dual-column fixation, bicolumnar plating has shown promise in improving stability and outcomes.

Objectives: This study aimed to evaluate the functional and radiological outcomes of distal femur fractures treated with bicolumnar plating using the Knee Society Score (KSS) and the Modified Radiological Union Score of Tibia (RUST) at the three-month, six-month, and one-year follow-up.

Materials and methods: This retrospective observational study included 50 patients aged 20-60 years with closed distal femur fractures treated with bicolumnar plating at R.L. Jalappa Hospital and Research Center between May 2021 and April 2024. Functional outcomes were assessed using the KSS, and radiological union was evaluated using the Modified RUST score. Data were analyzed using IBM SPSS Statistics for Windows, Version 26.0 (Released 2018; IBM Corp., Armonk, NY, USA), with descriptive statistics and paired t-tests.

Results: Mean KSS progressively improved from 64.2 ± 2.8 at three months to 93.4 ± 2.2 at one year (t = 48.53, p < 0.001). Similarly, Modified RUST scores improved from 17.6 ± 1.8 to 27.3 ± 1.3 (t = 39.72, p < 0.001). Five (10%) patients experienced complications. The improvement in both outcome measures was statistically significant (p < 0.05).

Conclusion: Bicolumnar plating is an effective method for managing complex distal femur fractures, offering reliable functional recovery and radiological union with a low complication rate.

## Introduction

Distal femur fractures constitute 3%-6% of all femoral fractures and exhibit a bimodal distribution: high-energy trauma in younger patients and low-energy mechanisms, such as falls, in the elderly [1,2]. These fractures pose significant challenges due to comminution, intra-articular involvement, and difficulty achieving stable fixation in osteoporotic bone [3].

Conservative treatment often led to stiffness, malunion, and prolonged immobility, prompting the evolution of internal fixation methods. Various implants, including single lateral locking plates, intramedullary nails, and dynamic condylar screws, have been used [4-6]. However, in highly comminuted or unstable fractures, single lateral plating may fail to provide adequate stability, resulting in nonunion or varus collapse [7,8].

Bicolumnar (dual) plating offers enhanced biomechanical stability by supporting both medial and lateral columns, making it particularly suitable for complex AO Type C fractures and osteoporotic bone [9,10]. Recent studies suggest improved early mobilization, alignment, and union rates with this method [11-13].

This study aimed to evaluate the functional and radiological outcomes of distal femur fractures treated with bicolumnar plating, using the Knee Society Score (KSS) and the Modified Radiological Union Score of Tibia (RUST) at three months, six months, and one year postoperatively.

## Materials and methods

Study design 

This retrospective study was conducted at R.L. Jalappa Hospital and Research Center, Kolar, between May 2021 and April 2024. 

Study population and sample size

Patients who underwent bicolumnar plating for distal femur fractures were included in the study. 

The inclusion and exclusion criteria are listed in Table 1. Data retrieved from the patient's medical records were thoroughly analyzed with at least a one-year follow-up. A total of 50 patients who met the inclusion criteria were analyzed in this study.

**Table 1:** Inclusion and exclusion criteria

Criteria type	Specific criteria
Inclusion criteria	Age 20-60 years
	Closed distal femur fractures (AO Types A and C)
	Treated with bicolumnar plating
Exclusion criteria	Pathological fractures
	Osteomyelitis
	Polytrauma
	Open distal femur fractures

Study measures

This study aimed to assess radiological union times in distal femur fractures fixed with bicolumnar plating using the Modified RUST score and analyze functional outcomes using the KSS to determine mobility, stiffness, and pain improvement.

Follow-up intervals

Evaluations were conducted at the third month, sixth month, and one year postoperatively.

Ethics statement

Ethical clearance was obtained from the Central Ethics Committee of Sri Devaraj Urs Medical College (approval number SDUMC/R&D/CEC/SDUCMC-PG/91/NF/-2025-26) to conduct the research. 

Statistical analysis

Data were analyzed using IBM SPSS Statistics for Windows, Version 26.0 (Released 2018; IBM Corp., Armonk, NY, USA). Continuous variables were expressed as mean ± standard deviation. The paired t-test was used to compare the KSS and Modified RUST scores at different postoperative follow-up intervals (three months, six months, and one year). The paired t-test was chosen because the same group of patients was assessed repeatedly over time, and this test appropriately accounts for within-subject correlations while minimizing inter-individual variability. It provides higher statistical power than independent tests when evaluating changes in outcomes across multiple time points within the same cohort. A p-value of <0.05 was considered statistically significant.

## Results

A total of 50 participants were included in this study. Table 2 presents the demographic characteristics of the study population. The mean age of participants was 39.4 ± 8.7 years. The sample consisted of 30 (60%) males and 20 (42%) females. In this study, 33 (66%) participants sustained fractures resulting from road traffic accidents and 17 (34%) resulting from falls.

**Table 2:** Patient demographics (n = 50)

Variable	Value
Total patients (n = 50)	n (%)
Mean age	39.4 ± 8.7 years
Gender	
Male	30 (60%)
Female	20 (40%)
Mechanism of injury	
Road traffic accidents	33 (66%)
Falls	17 (34%)

Functional recovery, assessed using the KSS score, showed a steady improvement throughout the study period. At three months, the mean KSS score was 64.2, indicating moderate impairment in joint function, stiffness, and pain. By six months, the score improved to 78.1, reflecting better mobility and reduced stiffness. At one year, the mean KSS score reached 93.4, demonstrating significant functional recovery, enhanced joint mobility, and improved weight-bearing ability (Table 3).

**Table 3:** Functional outcomes of the study participants using the KSS score (n = 50) KSS: Knee Society Score. The paired t-test was used, and significance was set at a p < 0.05.

Time point	Mean ± SD	Min	Max	Test statistic	p-value
Three months	64.2 ± 2.8	60	69	t = 48.53	p < 0.001
Six months	78.1 ± 5.2	70	89		
One year	93.4 ± 2.2	90	97		

Radiological assessment using the Modified RUST score showed a progressive increase in bone healing over the follow-up period. By three months, the mean score was 17.6, indicating advancement of the biological healing cascade with emergent callus formation. At six months, the mean score was 22.8, showing significant improvement, representing consolidation and remodeling phases of fracture healing. And by one year, significant improvement was observed, with a mean of 27.3, showing bone union (Table 4).

**Table 4:** Radiological outcomes of the study participants using the Modified RUST score (n = 50) RUST: Radiological Union Score for Tibia. The paired t-test was used, and significance was set at a p < 0.05.

Time Point	Mean ± SD	Min	Max	Test statistic	p-value
Three months	17.6 ± 1.8	15	20	t = 39.72	p < 0.001
Six months	22.8 ± 1.4	20	25		
One year	27.3 ± 1.3	25	30		

In our study, five (10%) patients developed complications, such as infection (2, 4%), knee stiffness (2, 4%), and non-union (1, 2%) (Table 5).

**Table 5:** Complications (n=50)

Complication type	n	%
Superficial infection	2	4
Knee stiffness	2	4
Non-union	1	2

## Discussion

The current study supports the advantages of bicolumnar plating in the management of complex distal femur fractures. The significant and progressive improvement in both functional (KSS) and radiological (Modified RUST score) parameters highlights the clinical efficacy of this method.

Our results showed that one-year postoperative KSS and RUST scores significantly improved compared to earlier follow-up intervals, with minimal complications. This is consistent with findings from Tsegaye et al., who reported that early fixation and stable constructs led to favorable outcomes in their Ethiopian cohort [1]. Similarly, Saini et al. noted that distal femur locking compression plates provided excellent results in 63.3% of their patients, with union achieved in all cases by six months [2]. Our study adds further support to these findings, particularly by using a dual plating approach that likely augments medial column stability.

Amin et al. demonstrated that lateral plating alone could result in excellent to good outcomes in over 75% of cases, but they reported higher incidences of varus collapse in comminuted fractures [3]. In contrast, our approach, which included medial column support via bicolumnar plating, was associated with no mechanical failures and only one case of delayed union, underscoring the construct’s mechanical advantage.

Moreover, the incidence of complications in our study was low (10%), which is favorable when compared to Lemsanni and Najeb, who reported superficial infection in 3.3% and DVT in 2.1% of cases treated with dynamic condylar screw systems [4]. The lower complication rate observed in our study may be attributed to standardized postoperative protocols, stringent infection control measures, and early initiation of mobilization.

Biomechanically, dual plating distributes stress across both femoral columns, reducing the likelihood of implant failure in osteoporotic or comminuted bone. Ricci et al. also emphasized the importance of maintaining alignment and length, noting that failure to do so in single-plate constructs often led to suboptimal outcomes [9]. Henderson et al. also raised concerns about healing complications with lateral locking plates alone, especially in distal femur fractures with poor bone quality [14]. In line with that, our study supports that medial plating provides essential support, especially in AO Type C fractures.

From a functional standpoint, early knee mobilization is vital for recovery. In our cohort, improvement in range of motion and patient-reported functional outcomes were noticeable as early as the six-month follow-up. These results align with those of Schütz and Südkamp, who highlighted early mobilization as a crucial factor in avoiding knee stiffness [10].

Recent meta-analyses suggest that dual plating may offer superior healing potential in complex fractures compared to single lateral plating, particularly in cases involving significant medial comminution or poor bone stock [15]. This further validates our findings and reinforces the growing consensus supporting bicolumnar strategies.

Griffin et al. compared intramedullary nailing and locking plate systems, finding similar functional outcomes but higher malalignment rates in nailing groups [6]. Given that our study focused on bicolumnar plating, it stands as a viable and perhaps superior option in unstable or intra-articular fractures.

Overall, our findings support the adoption of dual-column fixation, particularly in complex and comminuted distal femur fractures, due to its superior stability, earlier return to function, and lower complication rates. However, our study is limited by its retrospective design, single-center experience, and relatively short follow-up. Future prospective multicenter studies with larger cohorts are needed to validate these findings.

Limitations

This study’s retrospective design introduces inherent limitations, such as potential selection and information bias, as data were obtained from existing medical records rather than a controlled prospective protocol. Additionally, confounding factors like variations in rehabilitation compliance or comorbidities could not be fully controlled. The relatively short follow-up duration of one year may not capture late complications, such as post-traumatic arthritis, implant failure, or long-term functional decline. Future prospective multicenter studies with longer follow-up periods and standardized rehabilitation protocols are recommended to validate and expand upon these findings.

## Conclusions

Bicolumnar plating is a reliable and effective method for treating distal femur fractures, demonstrating significant improvements in both functional and radiological outcomes over a one-year follow-up. However, given the retrospective nature of this study and the limited sample size, the results should be interpreted with caution to avoid overgeneralization. The findings provide supportive evidence for the biomechanical advantages of dual-column fixation, but long-term, prospective, multicenter studies are warranted to confirm these outcomes and evaluate durability, late complications, and patient-reported measures over extended follow-up periods.
